# What drives mixed-species shoaling among wild zebrafish? The roles of predators, food access, abundance of conspecifics and familiarity

**DOI:** 10.1242/bio.059529

**Published:** 2023-01-23

**Authors:** Ishani Mukherjee, Anuradha Bhat

**Affiliations:** Department of Biological Sciences, Indian Institute of Science Education and Research Kolkata, Mohanpur, West Bengal 741246, India

**Keywords:** Mixed-species groups, Wild zebrafish, Tropical fish, Shoaling, Predator risk

## Abstract

Mixed-species groups occur across a wide range of faunal communities and provide several benefits to members. While zebrafish have often been observed to form mixed-species shoals with coexisting species, the factors determining their occurrence are not yet fully understood. Shoals comprising zebrafish (Danio rerio), flying barbs (Esomus danricus), and whitespots (Aplocheilus panchax) were collected from a stagnant canal at Haringhata (West Bengal, India), and using laboratory-based experiments, we deciphered likely drivers of mixed-species shoaling among zebrafish. Experiments assessing foraging efficiency revealed that the amount of food consumed by individual zebrafish in mixed shoals was comparable to the amount consumed by these individuals in conspecific shoals. Within mixed-species shoals, zebrafish individuals, despite being smaller than the other species, consumed a comparable amount of food as the other species. Shoal choice experiments revealed that under predator risk, zebrafish associate more with mixed shoals and showed comparable associations to shoals differing in the abundance of conspecifics. Furthermore, zebrafish preferred associating with familiar conspecifics over unfamiliar mixed and unfamiliar conspecific shoals. Therefore, equitable food consumption in mixed shoals, greater association with mixed shoals in the presence of predators, and familiarity were important in driving zebrafish towards mixed-species shoaling.

This article has an associated First Person interview with the first author of the paper.

## Teaser

**Summary:** In their natural habitats, zebrafish often shoal with co-occurring species. This study reveals that food apportionment, benefits related to predator avoidance and familiarity are potential drivers towards mixed-species shoaling among zebrafish.

## INTRODUCTION

Predator avoidance and the need to forage efficiently are the main drivers of group living across a wide range of taxa (Rubenstein, 1978). The cohesion and longevity of group formation in organisms vary based on a variety of factors like sex-composition, predation pressure and nutrient availability (Bon et al., 1990; Ward and Webster, 2016; Herbert-Read et al., 2017; Ioannou, 2021). These can also further depend on age, species composition, and personality types of members within these groups (Ward et al., 2002; Goodale et al., 2020). Mixed-species groups comprise individuals belonging to more than one species, in spatial proximity to each other and interacting with one another to maintain the group (Goodale et al., 2017). Such groups often confer more benefits as compared to single species groups of comparable sizes. (Goodale et al., 2017; Stensland et al., 2003). Multi-species biofilms comprising many different species of microorganisms have greater resistance to certain antimicrobials as compared to the ones comprising a single species (Lee et al., 2014). Several bird species form multi-species flocks as these provide group members with enhanced anti-predator and foraging benefits (Hino, 2000; Sridhar et al., 2009; Sridhar et al., 2013). Mixed-species associations have been observed involving two monkey species (*Cercopithecus diana* and *Cercopithecus campbelli*)*,* where both species benefit from the association in terms of improved foraging and increased social behavior (Wolters and Zuberbühler, 2003).

Most studies related to shoaling in fishes focus on single species shoals and few consider mixed shoals (Paijmans et al., 2019). Despite this, there are some interesting findings so far: while certain species increase food intake by forming mixed-species shoals, others form mixed shoals to avoid a predator (Lukoschek and McCormick, 2000; Mathis and Chivers, 2003). The Indo-pacific Sergeant fish (*Abudefduf vaigiensi*) form mixed-species groups to obtain both these benefits i.e., to reduce the chances of predation as well as to increase food access (Paijmans et al., 2020). Mixed-species shoals comprising two species of sticklebacks (three-spined stickleback: *Gasterosteus aculeatus* and nine-spined stickleback: *Pungitius pungitius*) are as cohesive as shoals of either stickleback species (Ward et al., 2018). Mixed-species shoals are also formed based on familiarity, lack of conspecifics, advantages related to social learning and passive mechanisms such as comparable swimming speed across species (Krause et al., 1999; Paijmans et al., 2019).

Zebrafish (*Danio rerio*) has been a popular model organism for studies on developmental biology, genetics and cell biology. More recently, the behavior and ecology of wild zebrafish has also been widely studied (Engeszer et al., 2007; Spence et al., 2008). Wild zebrafish are found in freshwater streams and ditches across India, Bangladesh and Nepal and a variety of shoal sizes (2-300 individuals) have been reported in the wild (Pritchard et al., 2001; Suriyampola et al., 2016). In their natural habitat, zebrafish have been observed to shoal with co-occurring species such as flying barbs (*Esomus danricus*), whitespots (*Aplocheilus panchax*)*, Rasbora daniconius* and *Puntius* spp. (Miller and Gerlai, 2011; Spence et al., 2008; Suriyampola et al., 2016). Even as mixed-species shoals are common across a range of zebrafish habitats, shoal properties can vary between populations based on ecological factors like water flow, vegetation and predation pressure (Suriyampola et al., 2016).

**Fig. 1.**
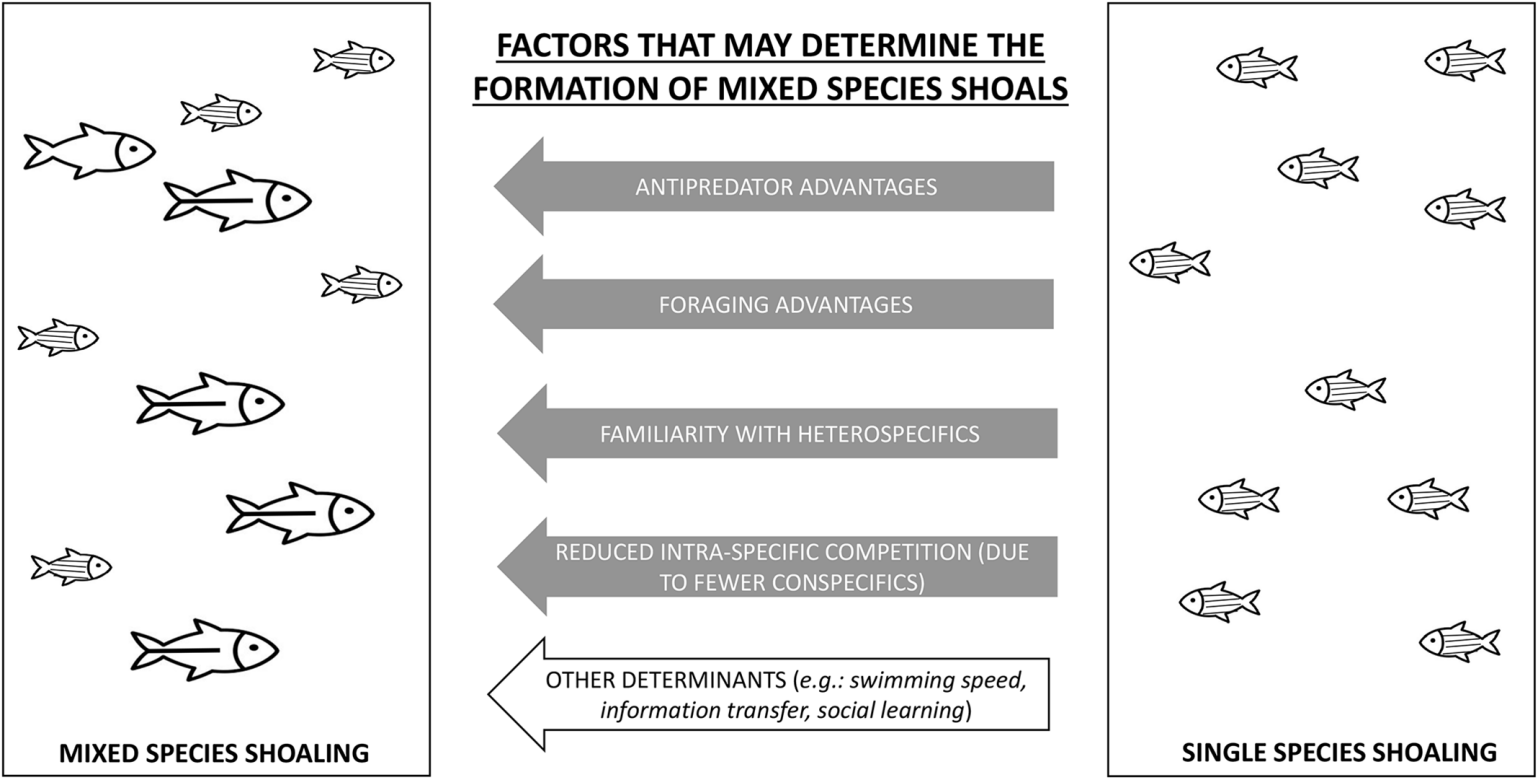
**Schematic showing the potential drivers that are likely to drive zebrafish towards mixed shoaling behavior.** While the drivers we test in our experiments have been highlighted in grey arrows, other likely drivers have been shown in a white arrow.

In a shallow and stagnant ditch at Haringhata (West Bengal, India) we observed mixed shoals comprising zebrafish, flying barbs and whitespots*.* These mixed shoals were collected and brought to the laboratory. Here, using laboratory-based experiments, we aim to explore four ecological factors that are likely to be important in driving wild zebrafish towards mixed-species shoaling. Specifically, we investigated whether benefits related to (1) foraging, (2) predator avoidance, (3) lack of conspecifics and (4) familiarity are likely to be important factors contributing to the prevalence of mixed species shoaling in wild zebrafish. The rationale for considering improved foraging as a potential driver towards mixed-species shoaling is as follows: it has been established that nutritional state of conspecifics drives shoaling decisions and foraging success of zebrafish shoals (Krause et al., 1999). Within mixed shoals, food might be easily accessible to zebrafish as the other species are known to not show aggression towards heterospecifics (Daniels, 2002). Thus, foraging benefits within mixed shoals might be a potential driver towards mixed shoaling in zebrafish. Another factor that controls shoaling in zebrafish is predation (Miller and Gerlai, 2011; Speedie and Gerlai, 2008) and in their natural habitat, zebrafish are prone to predation by the snakehead (*Channa* spp.) (Spence et al., 2008). Shoaling in mixed-species shoals can be a strategy to evade predation as fish predators often selectively prey on a particular species within such mixed shoals (Mathis and Chivers, 2003). *Channa* individuals might have a preference for the larger fishes (whitespots or flying barbs) within the same shoal and this might drive zebrafish towards mixed-species shoaling. We predict that to adhere to ‘safety in numbers’ strategy, zebrafish might shoal with other species when there is low abundance of conspecifics in the habitat. Familiarity is an important driver of inter-shoal associations and field-based studies have revealed that individuals form persistent associations with familiar individuals (Hay and McKinnell, 2002; Croft et al. 2004). Studies have shown that fish prefer shoaling with familiar heterospecifics over unfamiliar conspecifics (Ward and Hart, 2003). Therefore, we predict that zebrafish may form mixed shoals based on familiarity with other co-occurring species. We, thus, hypothesized that foraging and anti-predator benefits, familiarity with co-occurring heterospecific species and low relative abundance of conspecifics are likely to be factors that may determine the formation of mixed-species shoals (Fig. 1). We conducted foraging efficiency and choice-based experiments on wild-caught mixed-species groups of fishes to disentangle the roles of these probable drivers of mixed-species shoaling.

## RESULTS

### Body length and weight measurements

Zebrafish differed significantly from the other two species in terms of body length as well as weight. The body length of zebrafish (mean±s.e., 2.48±0.08 cm) was significantly less than flying barbs (mean±s.e., 3.86±0.09 cm) and whitespots (mean±s.e., 4.22±0.14 cm) (GLM: *n*_flying barb_=13, *n*_zebrafish_=32, *n*_whitespot_=11, Wald type IIχ 2=193.61, d.f.=2, *P*<0.0001; whitespot versus zebrafish: *Z* value=−12.06, *P*<0.0001, flying barb versus zebrafish: *Z* value=9.87, *P*<0.0001) (GLM details in Table S2). Zebrafish (mean±s.e., 0.26±0.03 g) weighed significantly less than flying barbs (mean±s.e., 0.51±0.02 g), which in turn weighed significantly less than whitespots (mean±s.e., 0.81±0.05 g) (GLM: *n*_flying barb_=22, *n*_zebrafish_=20, *n*_whitespot_=19, Wald type IIχ 2=115.22, d.f.=2, *P*<0.0001; zebrafish versus flying barbs: *Z* value=5.16, *P*<0.0001, zebrafish versus whitespot: *Z* value=−10.73, *P*<0.0001, whitespot versus flying barbs: *Z* value=−5.87, *P*<0.0001) (GLM details in Table S3).

### Foraging behavior of shoals from the standpoint of advantages to zebrafish

The average food consumption by zebrafish individuals was comparable when they were in conspecific shoals and in mixed-species shoals (low food treatments: χ^2^=0.004, d.f.=1, *P*=0.94, Fig. 3A; high food treatments χ^2^=0.17, d.f.=1, *P*=0.67; Fig. 3B). Within mixed shoals, the proportion of food eaten per individual per species was comparable across all three species (generalized linear model, GLM: Wald type IIχ 2=3.11, d.f.=2, *P*=0.21) and treatment types i.e., high food or low food conditions (GLM: Wald type IIχ 2=0.02, d.f.=1, *P*=0.87; Fig. 3C; Table 1).

**Fig. 2.**
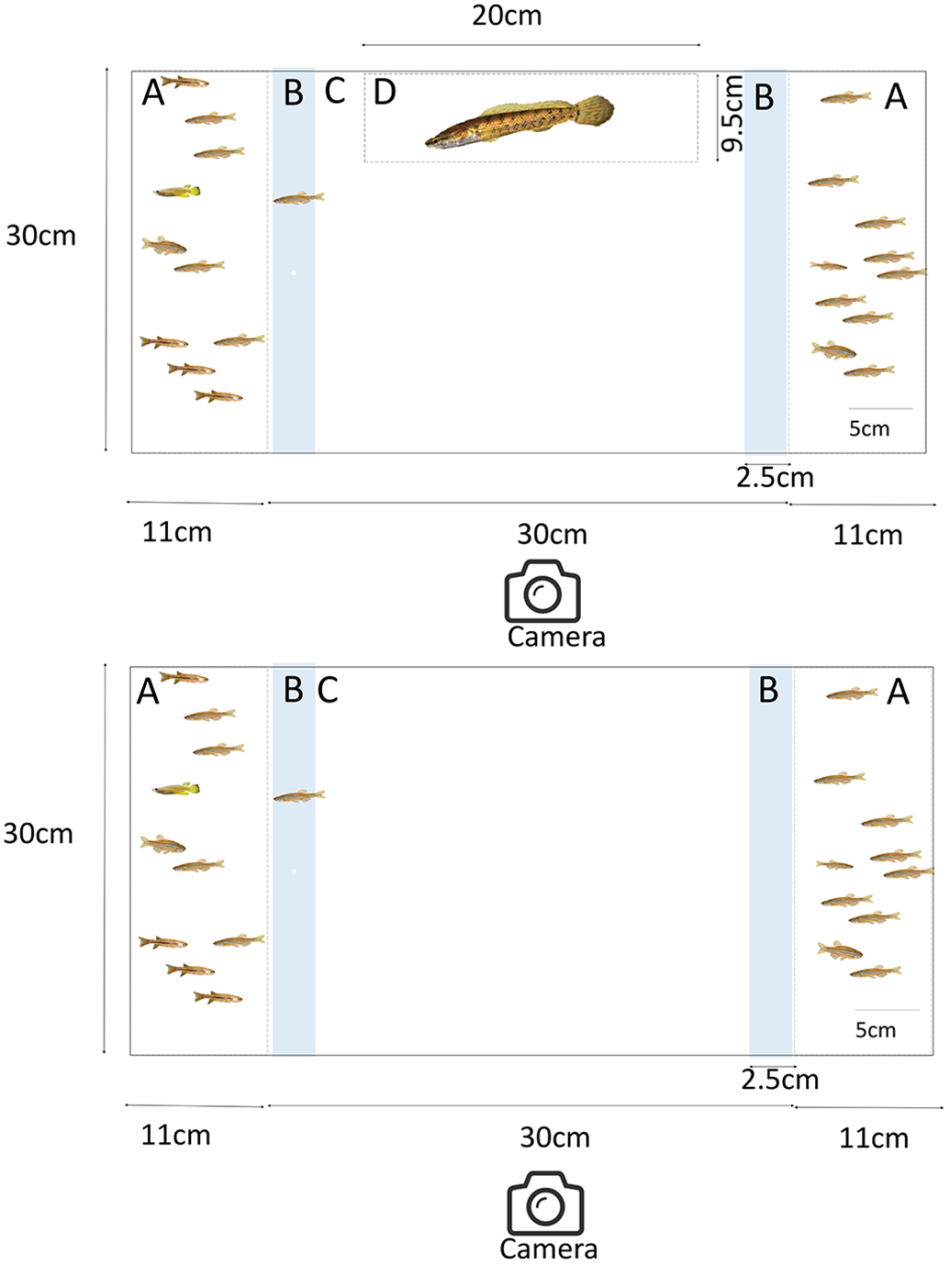
**Side view of the two-choice experiment tanks.** (A) The two-choice experimental tank where test fish are exposed to two shoals in the presence of a predator. (B) The two-choice experimental tank where test fish are exposed to two shoals in the absence of a predator. A: the end compartment that holds the stimulus shoals; B: association zone; C: central compartment; D: predator chamber. The schematics have been drawn to scale. Scale bar: 5cm.

**Fig. 3.**
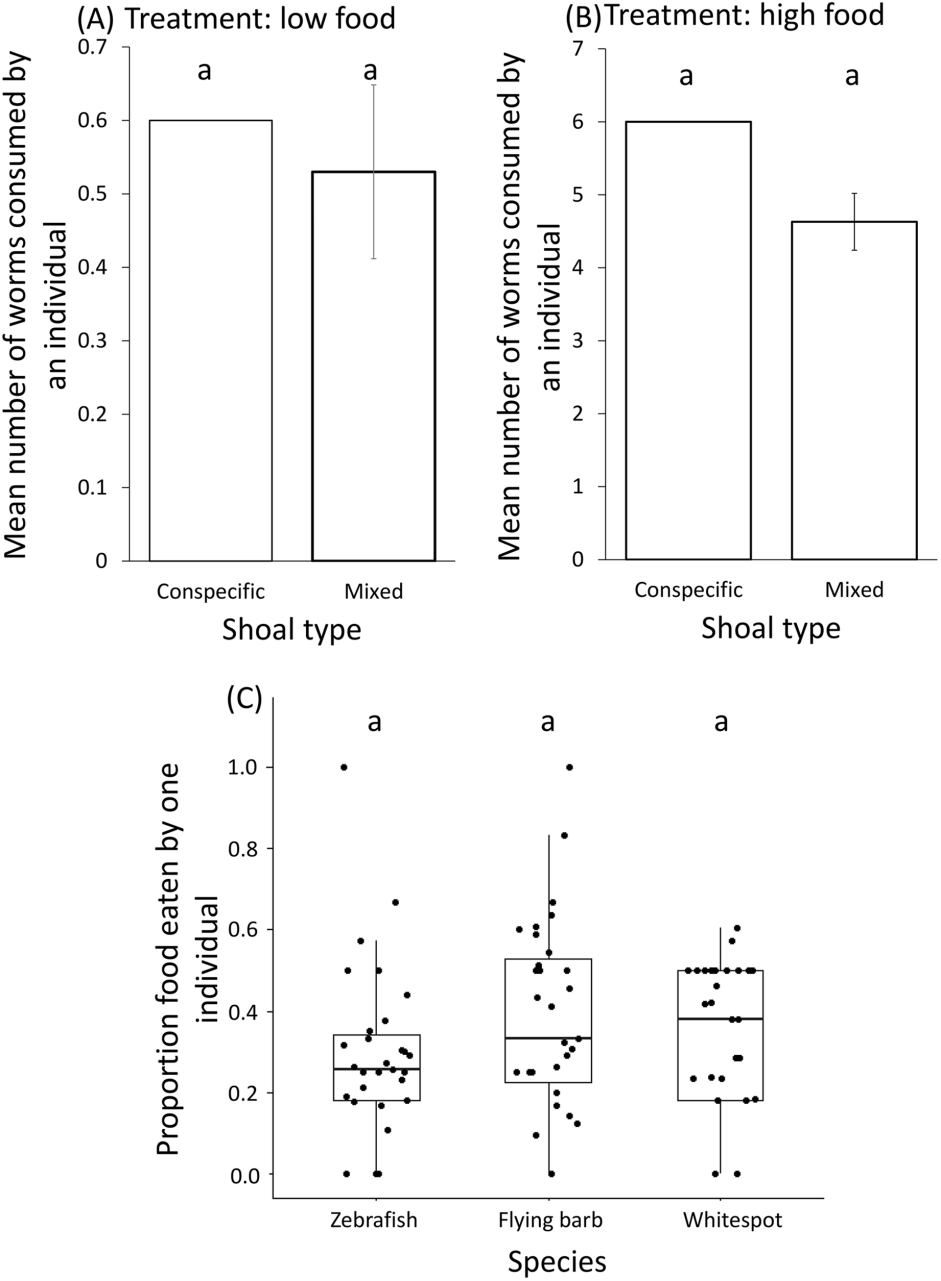
**Mean (±s.e.) amount of worm consumed by a zebrafish individual in conspecific shoal and in a mixed species shoal in (A) low food treatment and in (B) high food treatment.** In low food treatments, the standard error of mean number of worms consumed by an individual is 0.11 for mixed species shoals and 0 for conspecific shoals. In high food treatments, the standard error of mean number of worms consumed by an individual is 0.39 for mixed species shoals and 0 for conspecific shoals. (C) The proportion worms eaten per individual for different species within mixed species shoals. Data points are represented as dots. The different letters placed above the boxes represent significant differences between the categories. In A and B comparisons have been made by performing Chi-square test and in C comparisons have been made by performing GLM followed by Tukey's HSD Test (*n*=30 shoals, *P*<0.05).

**Table 1.**
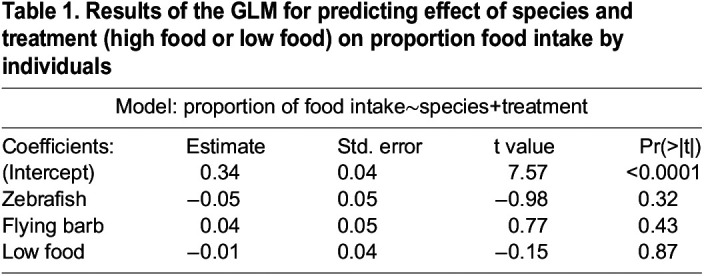
Results of the GLM for predicting effect of species and treatment (high food or low food) on proportion food intake by individuals

In low food as well as high treatments, there was a significant effect of shoal composition on feeding time (low food treatments: GLM: *n*_zebrafish_=15 shoals, *n*_flying barb_=14 shoals, *n*_whitespot_=13 shoals, *n*_mixed_=15 shoals, Wald type IIχ 2=13.89, d.f.=3, *P*<0.01; Tables S4 and S5; Fig. S2A; high food treatments: GLM: *n*_zebrafish_=*n*_whitepot_=*n*_mixed_=15 shoals, *n*_flying barb_=16 shoals, Wald type IIχ 2=23.11, d.f.=3, *P*<0.0001; Tables S6 and S7; Fig. S2B). While the feeding time of zebrafish shoals (mean±s.e., 5.06±1.28 s) in low food treatments was significantly lower than whitespot shoals (mean±s.e., 13±2.13 s) (zebrafish versus whitespot: *Z* value=−3.58, *P*<0.01), in high food treatments the feeding time of whitespot shoals (mean±s.e., 94.8±16.3 s) was significantly lower than that of zebrafish (mean±s.e., 216.6±18.12 s) (whitespot versus zebrafish: *Z* value=4.75, *P*<0.001; whitespot versus flying barb: *Z* value=2.77, *P*=0.02). Among the three species, zebrafish (mean±s.e., 0.46±0.05 mgl^−1^ h^−1^) consumed significantly lesser oxygen as compared to whitespot (mean±s.e., 0.69±0.05 mgl^−1^ h^−1^) (GLM: *n*_zebrafish_=*n*_flying barb_=28, *n*_whitespot_=19, Wald type IIχ 2=8.31, d.f.=2, *P*=0.01; Tables S8 and S9; Fig. S2C).

### Shoaling preference in presence of a predator

**Fig. 4.**
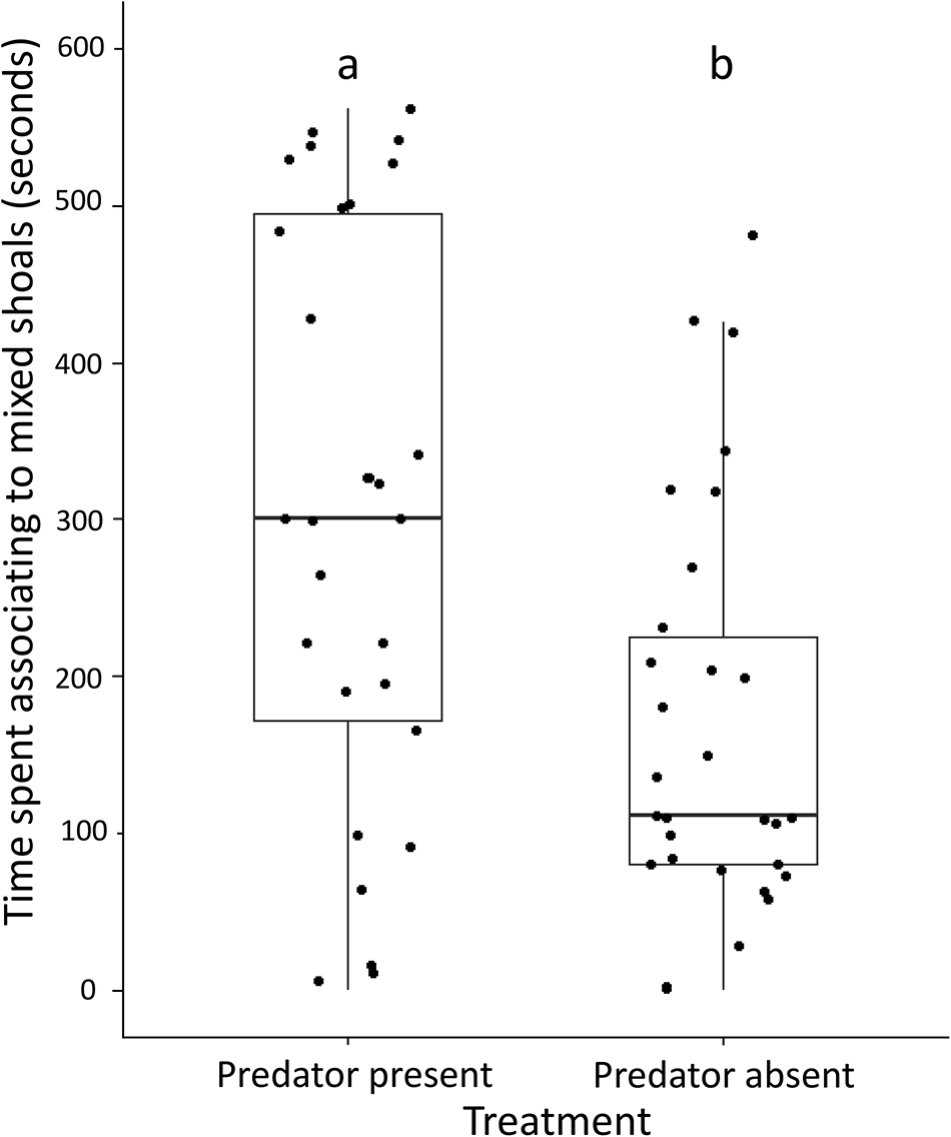
**Time spent by test zebrafish in association with mixed shoals, in the presence and absence of a predator.** Data points are represented as dots. The different letters placed above the boxes represent significant differences between the categories. The comparisons have been made by performing Wilcoxon unpaired test (*n*=30 shoals, *P*<0.05).

Test zebrafish associated with a mixed shoal significantly more in the presence of a predator (mean±s.e., 297.2±33.61 s) than in absence of a predator (mean±s.e., 169±23.19 s) (*W*=270.5, *n*=30, *P*<0.01; Fig. 4). Further, in treatments where the predator was present, the number of visits by test individuals to the mixed shoal was comparable between the first 2 min (mean±s.e., 2.66±0.48) and last 2 min (mean±s.e., 2.73±0.38) of the recording (*V*=108.5, *n*=30, *P*=0.81). We also conducted experiments where test predators (snakehead individuals) were given a choice between a zebrafish shoal and a flying barb shoal. Although test snakeheads struck comparably at the zebrafish shoal (mean±s.e., 4.72±1.06 times) and flying barb shoal (mean±s.e., 4.90±1.06 times) (*V*=28, *n*=11, *P*=1), the first two strikes were significantly more towards flying barb shoals (towards zebrafish shoal: mean±s.e., 0.45±0.19 times; towards flying barb shoal: mean±s.e., 1.54±0.19 times) (*V*=4.5, *n*=11, *P*=0.04).

### Shoaling preference when the abundance of conspecifics varied

**Fig. 5.**
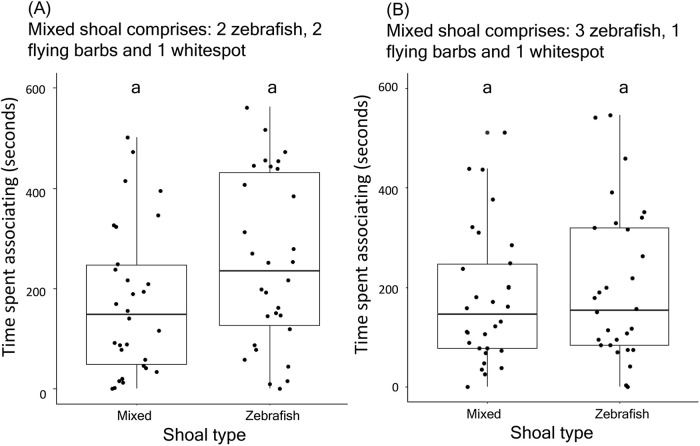
**Time spent by test zebrafish in association with: (A) conspecific shoals comprising five zebrafish and mixed shoals comprising two zebrafish, two flying barbs and one whitespot, and (B) conspecific shoals comprising five zebrafish and mixed shoals comprising three zebrafish, one flying barb and one whitespot.** Data points are represented as dots. The different letters placed above the boxes represent significant differences between the categories. The comparisons have been made by performing Wilcoxon unpaired test (*n*=30 shoals, *P*<0.05).

Test zebrafish showed similar preference for a conspecific shoal and a mixed shoal comprising different proportions of conspecifics, where the overall number of individuals in both shoals remained same. The association time with a conspecific shoal comprising five zebrafish (mean±s.e., 252±30.44 s) was comparable to association time with a mixed shoal comprising two zebrafish, two flying barbs and one whitespot (mean±s.e., 174.5±26.45 s) (*V*=295, *n*=30, *P*=0.21, Fig. 5A). Similarly, the association time with a conspecific shoal comprising five zebrafish (mean±s.e., 197.13±27.87 s) was comparable to association time with a mixed shoal comprising three zebrafish, one flying barb and one whitespot (mean±s.e., 178.13±24.22 s) (*W*=249.5, *n*=30, *P*=0.74; Fig. 5B). We found that test zebrafish individuals showed a preference for a conspecific shoal over heterospecific shoal (i.e. with only flying barbs and no zebrafish) (*V*=50.5, *n*=10, *P*=0.02).

### Familiarity as a factor in shoal association choice

When presented a choice between shoals varying in familiarity and species composition (i.e. conspecific shoals and mixed shoals), test zebrafish had a greater association time with familiar conspecific (FC) shoals (mean±s.e., 273.93±27.91) than unfamiliar mixed (UM) shoals (mean±s.e., 150.66±21.33) (FC versus UM: *V*=343, *n*=30, *P*=0.02) and FC shoals (mean±s.e., 241.9±40.96) than unfamiliar conspecific (UC) shoals (mean±s.e., 64.5±10.97) (*V*=3, *n*=10, *P*=0.02). Test zebrafish showed comparable associations with stimuli shoals in the other choices provided [familiar mixed (FM) versus UC: *V*=238, *n*=30, *P*=0.91; UM versus UC: *V*=212, *n*=30, *P*=0.68; FC versus FM: *V*=256, *n*=30, *P*=0.64; FM versus UM: *V*=27, *n*=11, *P*=0.62].

## DISCUSSION

The main aim of the study was to unravel the multiple roles of the presence of conspecific and co-occurring species, familiarity, and predation risk on the shoaling decisions among zebrafish. For this, we used a multipronged approach based on a variety of choice-based experiments to specifically understand the effect of these factors on shoal choice. Firstly, to investigate whether forming mixed-species shoals benefit zebrafish, we tested individuals in four different contexts. Our results indicate that food availability, protection from predators and familiarity together drive zebrafish towards shoaling with other species. We found that despite being smaller than the other species, zebrafish individuals consumed similar amounts of food to the other species within mixed shoals. Further, the amount of food consumed by individual zebrafish in mixed shoals and in single species (i.e. zebrafish-only) shoals were comparable. Shoal choice experiments revealed that zebrafish prefer a mixed shoal under predation threat and familiar shoals over unfamiliar shoals. Interestingly, the abundance of conspecifics did not impact their shoaling decisions. These results indicate that there are several advantages to forming mixed-species shoals, which may together provide an explanation for mixed-species shoaling decisions in zebrafish.

### Foraging behavior of shoals from the standpoint of advantages to zebrafish

Inter-species competition for food resulting in unequal foraging amongst species often occurs in mixed shoals (Mathis and Chivers, 2003; Lukoschek and Mccormick, 2000; Paijmans et al., 2020; Camacho-Cervantes et al., 2019). Contrary to these observations, in our study, we found that comparable amount of food was consumed by zebrafish individuals in single species and mixed-species shoals. Furthermore, this finding was consistent in low as well as in high food conditions. This suggests that regardless of the amount of food available, interspecies competition for food within these tropical shoals comprising zebrafish, flying barbs and whitespots is similar to that in conspecific shoals. Within mixed shoals, despite being significantly smaller than both species and having a lower metabolic rate than whitespots, zebrafish consume a similar amount of food to the other two species. There are two likely reasons for this. Firstly, whitespots, the largest of the species, are believed to show non-aggressive behavior towards other species (Daniels, 2002) and this might enable zebrafish (despite being smaller than the other species) as much access to food as the other two species. Secondly, reasonable abundance of plankton in tropical ponds and ditches means that inter- as well as intraspecific competition is unlikely to be very high in mixed shoals in these habitats. Thus, low or near-absent inter-species competition is an important likely factor that enables mixed-species shoaling and hence, foraging benefits in the form of equal access to food could be an important determinant favoring shoaling association of zebrafish with heterospecifics.

Despite inter-species food apportionment in mixed-species shoals, there was a difference in foraging time between shoals comprising only zebrafish and shoals comprising only whitespots. It is likely that these differences are a result of differences in size, oxygen intake (thus, metabolic rate), mouth types and feeding habits. While we report differences in body size and oxygen consumption, morphological trait parameters have been characterized in previous studies: zebrafish mouths are obliquely directed upwards with a longer lower jaw and these fish are surface as well as column feeders. Whitespots, on the other hand, have a small protrusible mouth (personal observation by the authors) and are chiefly surface feeders (Das et al., 2010; Spence et al., 2006). It is likely that under high food conditions, zebrafish tend to get satiated and thus spend a longer time feeding than whitespots (which, being larger and having a greater metabolic rate, were not satiated). The foraging time for mixed shoals was similar to that of single-species shoals. This suggests that even in mixed-species shoals, individuals did not deviate from their foraging patterns. Consistent with our findings, other experiments involving *Pseudomugil signifier* and *Gambusia holbrooki* show that foraging behavior is not plastic, and species are unable to adjust foraging behavior in mixed-species shoals (Keiller et al., 2021).

### Shoaling preference in presence of a predator

Apart from differences in body size, the three fish species also differ in their external morphology/appearance. Zebrafish have five uniformly pigmented horizontal stripes, while flying barbs have a single dark, broad lateral line (sometimes absent) and whitespots lack stripes but have a dark blotch at the lower third of the dorsal fin (Das et al., 2010). Interestingly, results on shoal choice experiments in the presence of a predator indicated that, test zebrafish spent significantly more time with a mixed shoal (comprising dissimilar individuals) as compared to a single species (conspecific) shoal. Thus, dissimilarity in size and phenotype does not play a role in shoaling decisions under predator threat i.e., oddity does not appear to control shoaling decision. In contrast to our findings, size segregation in shoals (to minimize oddity) often occur in the presence of a predator: mixed cyprinid shoals and shoals comprising certain minnow species segregate in accordance with size in the presence of a predator (Hoare et al., 2000; Allan and Pitcher, 1986; Theodrakis, 1989). In laboratory tests where the predator was given a choice between a flying barb shoal and a zebrafish shoal, starved *Channa* individuals were found to strike at flying barb shoals more often than zebrafish shoals. Therefore, we speculate that the preferred association of test zebrafish to mixed shoals could be due to the predators’ preference for whitespots and flying barbs over zebrafish. Across taxa, predators selectively favor prey species based on size (body length) and body mass (Owen-Smith and Mills, 2008; Dalal et al., 2020; Prado et al., 2020). Flying barbs and whitespots are larger in size and slower swimmers as compared to zebrafish (personal observations by the authors), and thus, for an ambush predator like the *Channa*, catching these species is more beneficial (in terms of net energy gained). Armored brook spined sticklebacks (*Culaea inconstans*) associated more with non-armored minnow shoals (*Pimephales promelas*) than with conspecific shoals. This is because the predator (perch) selectively attacked minnows in mixed shoals, thereby reducing the vulnerability of the three spined sticklebacks (Mathis and Chivers, 2003). Apart from differential preference by the predator, mixed shoals might also provide higher vigilance (using heterospecific alarm cues) and could be an additional reason for mixed shoaling in the presence of predators (Ward et al., 2018). In consensus with our findings, damselfish (*Abudefduf vaigiensis*) associate equally with conspecific shoals and shoals of the phenotypically different Australian mado (*Atypichthys strigatus*), indicating that oddity is not a universal driver of shoaling decisions (Paijmans et al., 2021).

### Shoaling preference when the proportion of conspecifics varied

Contrary to expectations, we did not find evidence that the abundance of conspecific individuals was important for shoaling decisions. We found that individuals show equal preference to shoals differing in proportion of conspecifics. In agreement with other studies (Snekser et al., 2010; Saverino and Gerlai, 2008), we found that test zebrafish individuals showed a preference for conspecific shoals over heterospecific shoals (where only flying barbs were present). Shoaling with other species have disadvantages such as reduced mating opportunities and increased oddity. Yet, test zebrafish associate comparably with mixed-species shoals and conspecific shoals. We speculate that advantages of shoaling with other species is comparable to the disadvantages (reduced mating opportunities, increased oddity), and thus, zebrafish make shoaling decisions irrespective of abundance of conspecifics. Another possibility is that in natural environments, zebrafish are unable to distinguish heterospecifics from conspecifics and thus, shoals form when two or more individuals are in spatial proximity to one another. In natural environments, under turbid conditions and low visibility, visual cues may not be efficient. Chubs (*Leuciscus cephalus*) rely greatly on olfactory cues to distinguish conspecifics from heterospecifics (Ward et al., 2019). We speculate that in environments with water flow and turbidity, detection and differentiation of olfactory cues between co-occurring species might be harder. This might be an explanation for similar preference of test zebrafish for conspecific shoals and mixed-species shoals. Further studies testing sensory mechanisms in recognizing heterospecifics and conspecifics will provide some insight into the mechanisms of forming mixed-species shoals.

### Familiarity as a factor in shoal association choice

Our findings suggest that familiarity is not a key factor in zebrafish choosing to shoal in mixed-species groups. However, test zebrafish made decisions based on familiarity when presented a choice between conspecific shoals. Zebrafish individuals showed comparable association between conspecific and mixed shoals (when stimulus shoals are either familiar or unfamiliar i.e. FM versus FC and UM versus UC). We speculate that this occurs because associating with either shoal can be beneficial: associating with mixed shoals have benefits such as improved predator avoidance and food apportionment (as discussed in the previous sections). Associating with conspecific shoals can provide mating benefits, and better social communication. Further, individuals also showed comparable association between UC and FM shoals, and UM and FM shoals. We speculate that this occurs because shoaling with either shoal has an advantage. In contrast to our findings on comparable association between UC and FM shoals, test chub (*Leuciscus cephalus*) individuals preferred familiar heterospecific (minnow or *Phoxinus phoxinus*) shoals over unfamiliar conspecific shoals (Ward and Hart, 2003). Shoaling with mixed shoals confer anti-predator and foraging benefits. Shoaling with familiar individuals is advantageous because of better predator evasion and foraging (Griffiths et al., 2004), more stable dominance hierarchies (Höjesjö et al., 1998; Gómez-Laplaza, 2005) and quicker recovery from fear (Mathuru et al., 2017 preprint). Thus, across species, fishes show a preference for shoaling with familiar individuals (Binoy and Thomas, 2006; Brown, 2002; Ward and Hart, 2003) over unfamiliar shoals (Brown and Smith, 1994; Griffiths and Magurran, 1997). In our choice tests too, to infer FC versus UC preference among zebrafish individuals, we found that zebrafish associated significantly more with familiar conspecific shoals over unfamiliar ones. Test individuals also associate with FC shoals significantly more than UM shoals. We speculate that this is because combined disadvantages associated with unfamiliarity and mixed shoaling (decreased mating opportunities and unstable social hierarchies) is sufficient to drive association tendencies towards FC shoals.

It should be noted that our study pertains specifically to wild zebrafish populations with experience of mixed species shoaling. A study on aquaria raised zebrafish found that zebrafish individuals form stronger associations with conspecifics over heterospecifics (Saverino and Gerlai, 2008). This is possibly due to the fact that lab-raised zebrafish are raised in a completely different environment in which selective forces (e.g. predation, competition) are absent. Further, these fish were not exposed to other co-occurring species such as flying barbs or whitespots. Therefore, it is reasonable for in-house bred fish to prefer conspecifics over heterospecifics.

Across taxa, literature on mixed-species groups is scant (Paijmans et al., 2019) and while most studies focus on whether benefits related to either foraging or predation drive mixed-species shoaling, ours show how various ecological factors control shoaling decisions differently, thereby giving greater insight into the ultimate causes of shoaling among wild zebrafish. Our results indicate that predator avoidance benefits, reduced inter-specific competition for food and familiarity with heterospecifics are likely to be determinants of mixed species shoaling among wild zebrafish. In addition to these factors, mixed shoaling can also be favored as a consequence of factors such as the inability to distinguish conspecifics. Other factors such as heterospecific differences in social learning amongst the species (Paijmans et al., 2019) and passive mechanisms such as comparable swimming speeds and learning may determine association preferences of zebrafish individuals (Krause et al., 1999). Thus, net benefits arising from multiple trade-offs are considered as ultimate explanations for mixed species shoaling (Paijmans et al., 2019). By examining foraging behavior and through shoal choice tests we focus on whether these factors are important in driving zebrafish towards mixed shoaling. Our results show that from the perspective of foraging, although it is not necessarily an advantage to shoal with heterospecifics, it suggests that as there is little cost involved in terms of competition for food, if formation of mixed species shoals provides predator avoidance benefits, it would be a favored. Given the especially heterogenous and dynamic conditions in natural habitats, we do note that our study cannot predict the relative importance these mentioned factors. Further, these results (on shoaling decisions of individuals) do not completely answer questions about why zebrafish populations differ in the relative abundances in mixed shoals or why mixed shoaling evolved in this species. Several other factors such as co-operation in information sharing, comparable swimming speed between species as well as interactive effects of these ecological factors may drive mixed-shoaling behavior in this species. While our study focuses on benefits obtained by zebrafish, it would be interesting to conduct investigations on what drives flying barbs and whitespots towards mixed-species shoaling. We suggest that such advantages combined with a variety of ecological factors provide ultimate explanations for the existence of such mixed-species shoals in these habitats.

## MATERIALS AND METHODS

### Collection and maintenance of shoals

This study was conducted on a wild-caught zebrafish population collected from its natural habitat at Haringhata (Nadia District) in West Bengal, India. Mixed shoals comprising mostly zebrafish and flying barbs have been observed to be feeding and swimming together at the surface of a stagnant ephemeral ditch. Some shoals also comprise a few whitespots or juveniles of *Puntius* sp.. Though owing to high turbidity and moderate vegetation cover it was not feasible to quantify the exact shoal size and composition, qualitatively, these shoals typically comprised 5-20 individuals, mostly zebrafish and flying barbs, and sometimes whitespots as well. Shoals consisting of zebrafish, flying barbs*,* and some whitespots were collected using a drag net. The fishes were brought to the laboratory in aerated plastic bags and housed in mixed-species groups of 50 individuals per 60 cm×30 cm×30 cm glass aquarium tanks, supplied with aerators. The relative abundance of species in each tank was comparable to the ratio in which they occurred in the natural habitat, i.e. each glass tank housed 20 zebrafish, 20 flying barbs and 10 whitespots. Single species tanks (consisting of ∼50 individuals of the same species housed together) were also maintained separately and these individuals were used in experiments which involved single species. A temperature range of 24-26°C and a constant lighting condition of 12 h dark:12 h light was maintained in the holding room. Fishes were fed daily with freeze-dried bloodworms or brine shrimp *(Artemia* sp.). Experiments commenced after a 30-day acclimatization to laboratory conditions.

Snakeheads (*Channa* spp.) are common predators to these fishes and occur widely in the same habitats (Spence et al., 2008). *Channa punctatus* individuals (approximately 16-17.5 cm in length) were collected from the same ditch and housed separately in 60×30×30 cm tanks and fed daily with pellet food or fish that died of natural causes in the laboratory.

### Body length and weight measurements

After 2 weeks of acclimatization to laboratory conditions, the body length (distance from the tip of mouth to the base of caudal fin) of 32 zebrafish, 12 flying barbs and 11 whitespots was measured from photographs taken using ImageJ (Schneider et al., 2012). The fish (20 zebrafish, 22 flying barbs and 19 whitespots) were weighed to the nearest 0.1 mg using a digital balance and experiments commenced 48 h after the measurements were taken.

### Foraging behavior of shoals from the standpoint of advantages to zebrafish

After a 24-h period of starvation, a single species shoal comprising five conspecifics per shoal (zebrafish, flying barbs or whitespots), or a mixed-species shoal (comprising two zebrafish, two flying barbs and one whitespot) was introduced into a 30×20×20 cm glass tank. The proportion of each species in a shoal was decided based on their relative abundance in their natural habitats (personal observations by the authors). To simulate natural conditions where scanty or abundant food are likely to be found, freeze dried blood worms were provided in low [three worms (≈0.7 mg)] or high [30 worms (≈17.0 mg)] amounts. A study by Spence et al. (2006) has shown that zebrafish as well as the other species mostly reside and feed at the top of the water column. Recent stable isotope analysis studies have shown that dietary habits among surface and column feeding co-occurring species from similar aquatic habitats in West Bengal are broadly comparable (Mondal and Bhat, 2021). Thus, in our experiment, we provided a single kind of food. After a 2-min acclimation period following the introduction of the shoal into the test tank, the food (either low or abundant food) was introduced onto the water surface, at a random corner of the tank. The shoal was recorded using a digital video camera (Canon Legria HF R306) placed directly above the test arena, for 10 min. To check whether the foraging behavior of individual zebrafish change based on the shoal composition (i.e. single species or mixed species), we used the video recordings to calculate the average number of worms consumed by zebrafish individuals in single-species shoals and in mixed-species shoals. This was calculated separately for high and low food treatments. Secondly, we determined the proportion of worms consumed per individual of each species within mixed-species shoals. Thirdly, we calculated the foraging time or the time taken to consume three worms (i.e. low food treatments) or 30 worms (i.e. high food treatments) for each shoal. All scoring was performed manually by a single observer (IM) blind to the treatment type. In the low food condition, we tested 15 zebrafish shoals, 14 flying barb shoals, 13 whitespot shoals and 15 mixed shoals. In the high food condition, 15 zebrafish shoals, 16 flying barb shoals, 15 whitespot shoals and 15 mixed shoals were tested. Therefore, a total of 118 shoals (57 shoals in low food condition and 61 in high food condition) were tested. Fish within shoals were tested only once per food treatment condition.

Oxygen consumption of a fish is proportional to metabolism (Martins et al., 2011). In order to understand the relationship between foraging behavior and metabolism for each species, oxygen consumed (in mg/l) in one hour by individuals (of different species) was measured. A detailed methodology for these experiments are included in the supplementary informaton (Fig. S1).

### Shoaling preference in zebrafish in the presence of a predator

The experimental tank (30 cm×30 cm×62 cm) was divided into three compartments using two transparent perforated partitions (to enable visual and olfactory cue exchange). The compartments at the two ends (11 cm×30 cm×30 cm) were used for the stimulus shoals. While one stimulus shoal had 10 conspecifics (i.e. a zebrafish shoal), the other had five zebrafish, four flying barbs and one whitespot (i.e. a mixed shoal). The stimuli shoals were swapped every 10 trials to ensure there was no preference for a particular side in the tank. The central compartment (40 cm×30 cm×30 cm) comprised three zones: two association zones that were 2.5 cm (i.e. approximately one body-length distance) away from the end chambers, were demarcated by lines drawn on the wall of the tank and a middle zone. Test individuals were considered associating with a stimulus shoal if any part of their body was inside the association zone. A transparent and perforated chamber (20 cm×30 cm×9.5 cm) was attached to the wall of the middle zone. In half of the trials, this chamber contained a snakehead (*Channa punctatus*). The experimental tank was filled with aged water to 10 cm depth and the stimuli shoals and the predator (in half of the trials) were introduced. After a period of 10 min, a test fish was released in the middle compartment (Fig. 2A). The movement of the test fish was then recorded for 10 min using a digital video-camera (Canon Legria HF R306) placed near the side of the experimental tank. Association preferences (i.e. time spent in the left and right association zones) of the test individual for either of the stimuli shoals were quantified by an observer blind to the treatment type. A total of 30 zebrafish subjects were tested in the presence and 30 subjects were tested in the absence of the predator.

To check whether the predator had a prey species preference, an additional set of two-choice trials were conducted where 11 *Channa punctatus* individuals (previously starved for 48 h) were given a choice between two different prey species (zebrafish/flying barb) placed in two opposing chambers, with the *Channa* (predator) placed in the central arena. The prey towards which the first two strikes were made as well as the total number of strikes made in 20 min was noted down (Supplementary Information for details of the experiment).

### Shoaling preferences when the abundance of conspecifics varied in the stimuli shoals

Low abundances of conspecifics in the environment may drive species to shoal with heterospecifics (Paijmans et al., 2019). Here, by performing two-choice experiments, we investigated whether zebrafish associate with other species because of low abundance of conspecifics. The experimental arena described above (but without the predator chamber) was used to conduct the experiments (Fig. 2B). It is established that zebrafish prefer larger conspecific shoals over smaller conspecific shoals only when the smaller shoal have four or fewer individuals (Seguin and Gerlai, 2017). Based on this, we presented test fish a choice between shoals comprising five individuals: a conspecific shoal comprising five zebrafish, and a mixed shoal comprising either three zebrafish, one flying barb, one whitespot or two zebrafish, two flying barbs, and one whitespot. Therefore, we presented test zebrafish a choice between two similarly sized shoals where the number of conspecifics differed in the range it could detect. Thirty of each kind of trial (i.e. a total of 60) were performed. Additionally, to test whether zebrafish have a preference for conspecific shoals over heterospecific shoals, we performed 10 trials in which test zebrafish were given a choice between a conspecific shoal and a heterospecific shoal (flying barb shoals) were tested.

### Familiarity as a factor in shoal association choice

Familiarity is a strong driver of shoaling decisions (Catellan et al. 2019; Barber and Wright, 2001) and species have been reported to shoal with familiar heterospecifics over unfamiliar conspecifics (Ward and Hart, 2003). To examine the role of familiarity in shoal association preferences, we tested the choice of zebrafish individuals when presented with shoals differing in the extent of their familiarity to the test individual. In the present study, individuals were considered familiar when they belonged to the same population and had been maintained in the same stock tank for a period of at least 30 days in the laboratory. Fish raised in isolation for numerous generations are considered unfamiliar to one another (Martins et al., 2011). Based on this, ‘unfamiliar’ individuals for this experiment were collected from a similar shallow slow-moving stream (situated near Kharagpur, 180 km away from the location of the first population at Haringhata, in West Bengal, India) and were kept in a separate stock tank. This, thus, ensured that the test zebrafish had never encountered the unfamiliar individuals. Individuals from the two populations were not significantly different in terms of size (Table S1) or any other external morphological features such as coloration or pigmentation (personal observations by the authors).

The experimental arena for the experiment was the same as was used in the previous shoal choice experiment (Fig. 2B). We assembled four kinds of stimuli shoals [familiar conspecific (FC) shoals, familiar mixed (FM) shoals, unfamiliar conspecific (UC) shoals and unfamiliar mixed (UM) shoals]. For each type of experimental trial, any of these two stimulus shoals were placed in the end compartments. Thus, six combinations of trials and, a total of 141 trials were performed. Test zebrafish individuals had to choose between: (1) FC shoals versus FM shoals (*n*=30 trials), (2) FC shoals versus UC shoals (*n*=10 trials), (3) FC shoals versus UM shoals (*n*=30 trials), (4) FM shoals versus UC shoals (*n*=30 trials), (5) FM shoals versus UM shoals (*n*=11 trials) and (6) UC shoals versus UM shoals (*n*=30 trials). For experiments involving testing of choice between familiar conspecific FC shoals and UC shoals, fewer trials (*n*=10) were performed because it is well known that zebrafish prefer familiar over unfamiliar conspecifics (Gerlach and Lysiak, 2006) and we intended only to verify what is already well established. Further, for choice tests between FM shoals and UM shoals, some unfamiliar individuals were lost (i.e. due to death) from the other species (members of UM shoals). Thus, 11 choice test trials of this kind could be performed.

Guidelines outlined by the Committee for the Purpose of Control and Supervision of Experiments on Animals (CPCSEA), Ministry of Fisheries, Animal Husbandry and Dairying, Government of India were followed in all aspects of maintenance and experimentation. All experimental protocols followed here have been approved by the Institutional Animal Ethics Committee (IAEC) and guidelines of Indian Institute of Science Education and Research (IISER) Kolkata, Government of India (Approval number IISERK/IAEC/AP/2021/70).

### Statistical analysis

Statistical analyses were performed using R, version 4.0.2 (R Core Team 2020). GLM were built (using ‘glm’ function in R) to check the effect of species on individual specific traits (i.e. body-length, weight, oxygen consumption), the effect of species on proportion worms consumed and the effect of shoal composition on foraging time. Before developing the models, the distribution of the data was checked using the ‘fitdistr’ function (Delignette and Dutang, 2015). As our data was not close to any specifically defined distribution, we did not add a link function into our models. In such GLMs the individual traits (length, weight, or oxygen consumption), proportion worms consumed, or foraging time or were the dependent variables, and the species or treatment (shoal composition) were the fixed factors. All model comparisons were performed using ANOVA in ‘car’ package (Fox and Weisberg, 2019) and post hoc paired tests (Tukey's post hoc HSD Test) were carried out for comparing the effects of factors that were significant. Chi square tests were performed to check whether the average food consumption by zebrafish individuals in conspecific shoals is comparable to the average food consumption by zebrafish individuals in mixed-species shoals. To compare the differences in time spent associating with mixed-species shoals in the presence and absence of a predator we performed Wilcoxon unpaired tests. Further, Wilcoxon paired tests were performed to compare association time of test zebrafish individuals to shoals differing in the abundance of conspecifics or in familiarity and species composition. The cut-off level for significance was set as *P*<0.05 for all comparisons.

## Supplementary Material

10.1242/biolopen.059529_sup1 Supplementary information
