# Supplementary material for: What drives mixed-species shoaling among wild zebrafish? The roles of predators, food access, abundance of conspecifics and familiarity
Source: Biol Open. 2023 Jan 23;12(1):bio059529. doi: 10.1242/bio.059529 (PMC9915908; doi:10.1242/bio.059529)
Supplement: Supplementary information [file biolopen-12-059529-s1.pdf]

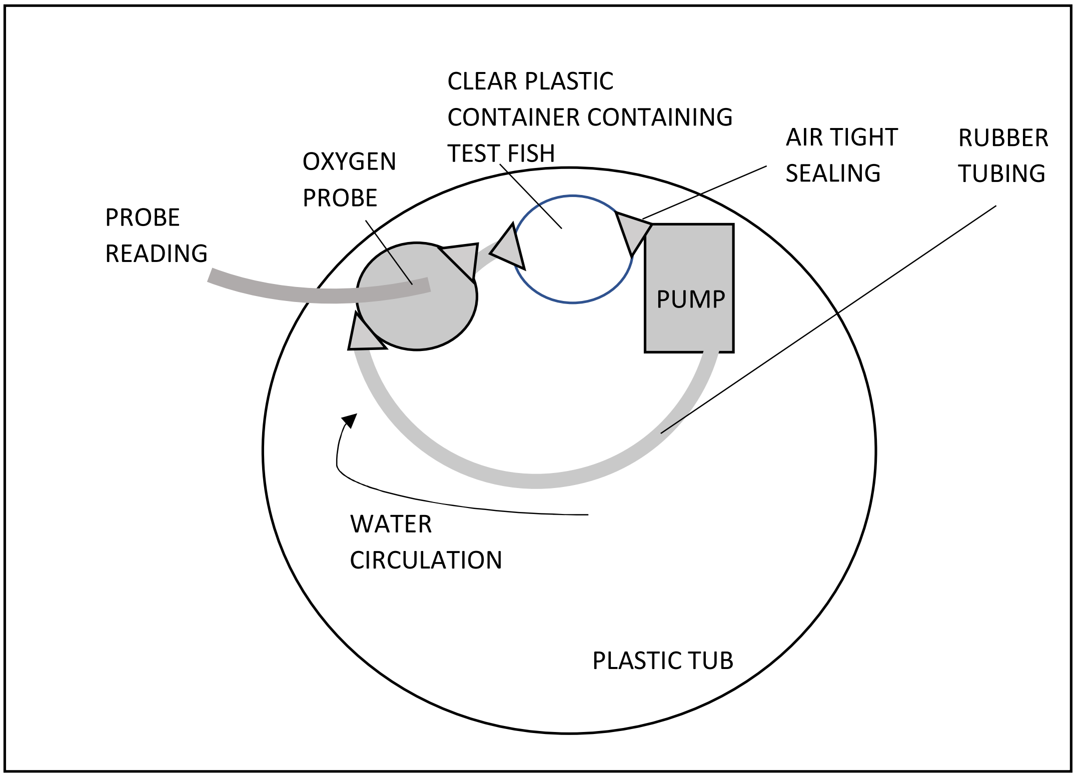

Fig. S1. Overhead view of the flow through respirometer set up (not to scale). The middle clear chamber (represented as white circle) fashioned from a plastic container (volume=200ml) contained a 24 hour starved test fish in post digestive state. An oxygen probe (Hanna HI 9142 Dissolved Oxygen Meter) that measured the dissolved oxygen concentration (represented as grey circle) was connected to the middle chamber. An electrical pump (represented as grey rectangle) constantly circulated water across the set up.

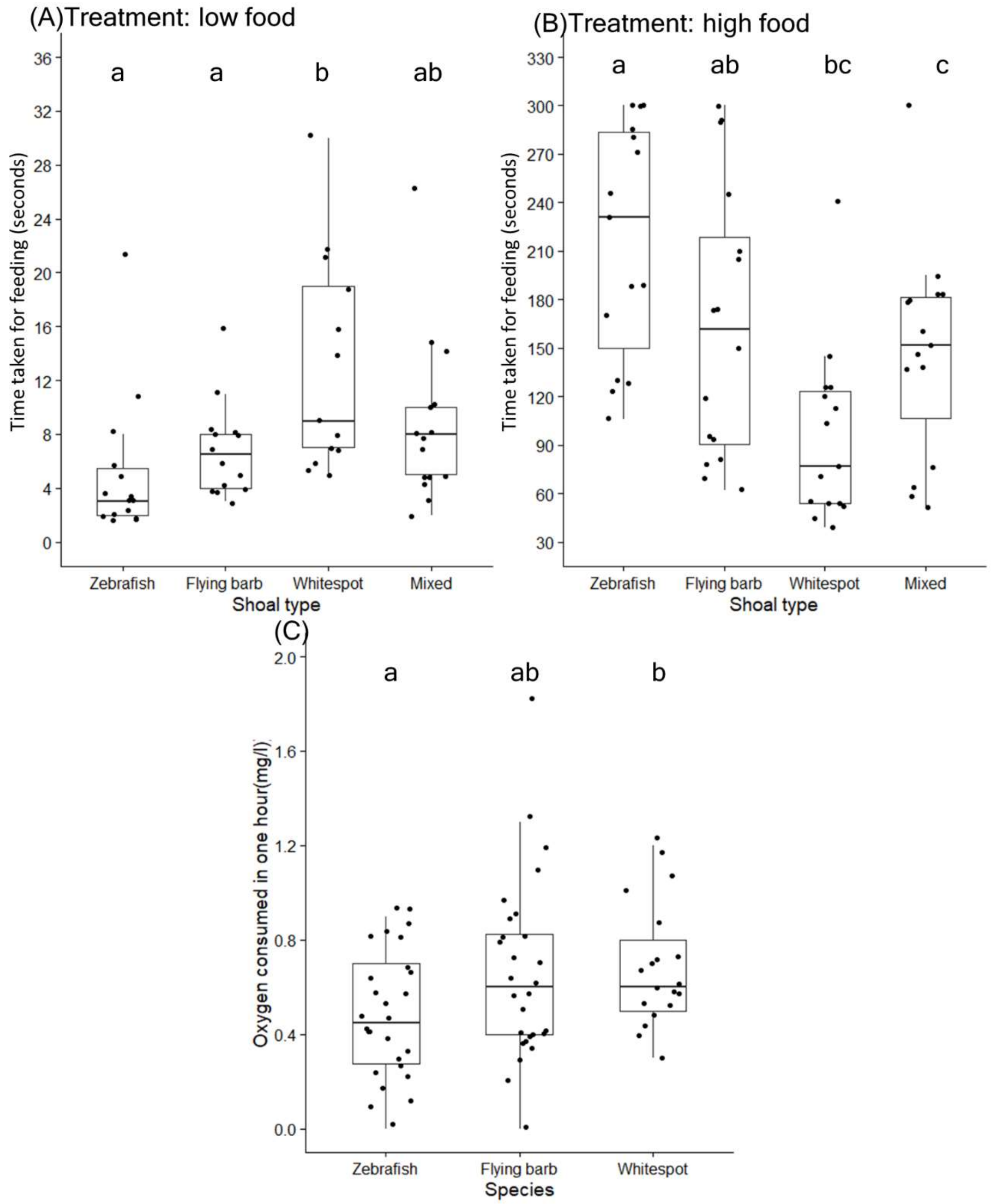

Fig. S2. Time taken for feeding (seconds) in (A)low food treatment and in (B) high food treatment. (C)The oxygen consumption (mg/l) in one hour for individuals across the three species. Data points are represented as dots. The different letters placed above the boxes represent significant differences between the categories. In A&B comparisons have been made by performing Chi-square test and in (C) comparisons have been made by performing GLM followed by Tukey's HSD Test ($p<0.05$).

Biology Open • Supplementary information

Table S1. Comparison of size between fish from Haringhata and fish from Kharagpur (used as stimuli shoals).

	Mean ± S.D. of body length of fish from Haringhata	Mean ± S.D. of body length of fish from Kharagpur	Wilcoxon unpaired test results
Zebrafish	2.48±0.41 cm	2.23±0.31	W = 91, p-= 0.21
Flying barbs	3.86±0.28cm	3.96±0.42	W = 59.5, p = 0.39

As only one whitespot individual (from Kharagpur) was used in the unfamiliar stimulus shoal, no test was conducted to compare whitespot sizes between the two populations.

Table S2. Comparison of body length of different species. Results of the generalized linear model (GLM) for predicting the effect of body length on association time of test fish.

Model: Body length ~Species

Coefficients:

	Estimate	Std. Error	t value	Pr(>|t|)
(Intercept)	4.21	0.12	33.89	<0.0001
Zebrafish	-1.74	0.14	-12.06	<0.0001
Flying barb	-0.35	0.17	-2.08	0.04

Table S3. Comparison of weight of different species Results of the generalized linear model (GLM) for predicting the effect of weight on association time of test fish.

Model: Weight ~Species

Coefficients:

	Estimate	Std. Error	t value	Pr(>|t|)
(Intercept)	0.81	0.04	22.10	<0.0001
Zebrafish	-0.55	0.05	-10.73	<0.001
Flying barb	-0.29	0.05	-5.87	<0.001

Table S4. Comparison of feeding time for low food conditions. Results of the generalized linear model (GLM) for predicting the effect of shoal composition on feeding time for low food treatments.

Model: Feeding time ~Shoal composition

Coefficients:

	Estimate	Std. Error	t value	Pr(>|t|)
(Intercept)	13.00	1.62	8.01	<0.0001
Zebrafish	-7.93	2.21	-3.58	<0.0001
Mixed	-4.33	2.21	-1.95	0.05
Flying barb	-6.14	2.25	-2.72	0.01

Biology Open • Supplementary information

Table S5. Comparison of feeding time for low food conditions. Tukey's HSD Test Results for estimating differences in feeding time between shoal types (i.e., zebrafish shoals, flying barb shoals, whitespot shoals and mixed shoals) under low food conditions.

Comparison	Z value	Pr(>|t|)
Mixed shoals Vs whitespot shoals	-1.95	0.20
Mixed shoals Vs zebrafish shoals	1.68	0.33
Mixed shoals Vs flying barb shoals	-0.83	0.83
Zebrafish shoals Vs flying barb shoals	0.82	0.84
Zebrafish shoals Vs whitespot shoals	-3.58	<0.01
Flying barb shoals Vs whitespot shoals	-2.72	0.03

Table S6. Comparison of feeding time for high food conditions. Results of the generalized linear model (GLM) for predicting effect of species on foraging time under high food conditions.

Model: Foraging time ~ Species

Coefficients:

	Estimate	Std. Error	t value	Pr(>|t|)
(Intercept)	94.80	18.11	5.23	<0.0001
Zebrafish	121.80	25.62	4.75	<0.0001
Mixed	52.00	25.62	2.03	0.04
Flying barb	69.95	25.21	2.77	<0.01

Biology Open • Supplementary information

Table S7. Comparison of feeding time for high food conditions. Tukey's HSD Test Results for estimating differences in feeding time between shoal types (i.e., zebrafish shoals, flying barb shoals, whitespot shoals and mixed shoals) under high food conditions.

Comparison	Z value	Pr(>|t|)
Mixed shoals Vs whitespot shoals	2.03	0.17
Mixed shoals Vs zebrafish shoals	-2.72	0.03
Mixed shoals Vs flying barb shoals	0.71	0.89
Zebrafish shoals Vs flying barb shoals	-2.05	0.16
Zebrafish shoals Vs whitespot shoals	4.75	<0.001
Flying barb shoals Vs whitespot shoals	2.77	0.02

Table S8. Results of the generalized linear model (GLM) for predicting effect of species and method (DO meter or in-house respirometer) on oxygen consumption by individuals.

Model: DO ~ Species + Method

Coefficients:

	Estimate	Std. Error	t value	Pr(>|t|)
(Intercept)	0.74	0.07	9.4	<0.0001
Zebrafish	-0.24	0.09	-2.58	0.01
Flying barb	-0.05	0.09	-0.53	0.59
Low food	-0.12	0.07	-1.57	0.11

Biology Open • Supplementary information

Table S9. Tukey's HSD Test Results for estimating differences in oxygen consumption between species (i.e., zebrafish, flying barbs, whitespots).

Comparison	Z value	Pr(>|t|)
Zebrafish Vs Whitespots	-2.58	0.02
Zebrafish Vs Flying barbs	-0.53	0.85
Whitespots Vs Flying barbs	2.29	0.05

Table S10. Comparison of snakehead's preference towards shoals differing in species composition.

	Towards zebrafish shoal	Towards flying barb shoal
Mean±S.E. number of total strikes	4.72±1.06 times	4.90±1.06 times
Wilcoxon paired test results of total number of strikes towards either shoal: V=28 , n=11, p = 1		
Mean±S.E. number of first two strikes	0.45±0.19 times	1.54±0.19 times

Wilcoxon paired test results of of first two strikes towards either shoal: V=4.5, n=11, p =0.04

Biology Open • Supplementary information
